# Supplementary material for: Disordered plasmonic system with dense copper nano-island morphology
Source: Nanophotonics. 2025 Apr 25;14(12):2151–60. doi: 10.1515/nanoph-2024-0743 (PMC12147547; doi:10.1515/nanoph-2024-0743)
Supplement: Supplementary file 1 — Supplementary Material Details [file j_nanoph-2024-0743_suppl_001.docx]

Research Article – SUPPORTING INFORMATION

Disordered plasmonic system with dense copper nano-island morphology

Tlek Tapani^1^, Vincenzo Caligiuri^2,3^, Yanqiu Zou^4^, Andrea Griesi^2^, Yurii P. Ivanov^2^, Massimo Cuscunà^5^, Gianluca Balestra^5^, Haifeng Lin^1^, Anastasiia Sapunova^2^, Paolo Franceschini^6^, Andrea Tognazzi^6^, Costantino De Angelis^7^, Giorgio Divitini^2^, Riccardo Carzino^2^, Hyunah Kwon^8^, Peer Fischer^8^, Roman Krahne^2^, Nicolò Maccaferri^1^ and Denis Garoli^2,9*^

^1^ Department of Physics, Umeå University, Linnaeus väg 24, 901 87 Umeå, Sweden

^2^ Istituto Italiano di Tecnologia, Via Morego 30, Genova, 16163 Italy

^3^ Dipartimento di Fisica, Università della Calabria, via P. Bucci 33b, 87036 Rende (CS), Italy.

^4^ College of Optical Science and Engineering, Zhejiang University, Hangzhou 310027, China.

^5^ Institute of Nanotechnology - CNR NANOTEC c/o Campus Ecotekne, Via Monteroni 73100 Lecce (Italy)

^6^ Università degli studi di Palermo, Dipartimento di ingegneria. Viale delle scienze ed. 10, 90128, Palermo, Italia

^7^ Università degli Studi di Brescia, Dipartimento di Ingegneria dell’Informazione, via Branze, 38

25123 Brescia, Italia

^8^ Institute for Molecular Systems Engineering and Advanced Materials, Heidelberg University, 69120 Heidelberg, Germany; Max Planck Institute for Medical Research, 69120 Heidelberg, Germany

^9^ Dipartimento di scienze e metodi dell’ingegneria, Università di Modena e Reggio Emilia, Via Amendola 2, 42122, Reggio Emilia, Italy

Email: [denis.garoli@unimore.it](mailto:denis.garoli@unimore.it)

**Supporting Note – 1**

**
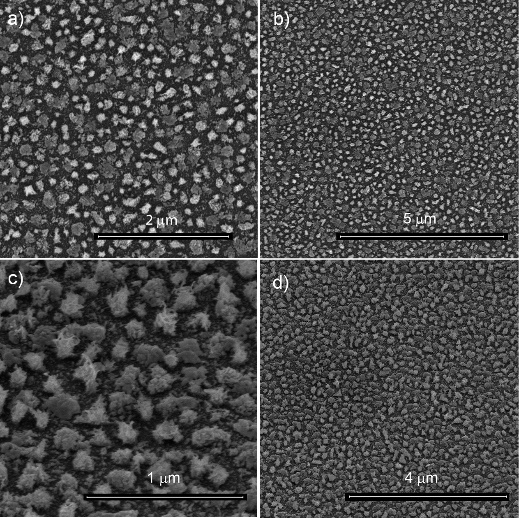
**

**Figure S1**. Additional SEM micrographs of the prepared Cu NIs film. Top view ((film obtained after two repeated evaporation+plasma etching).


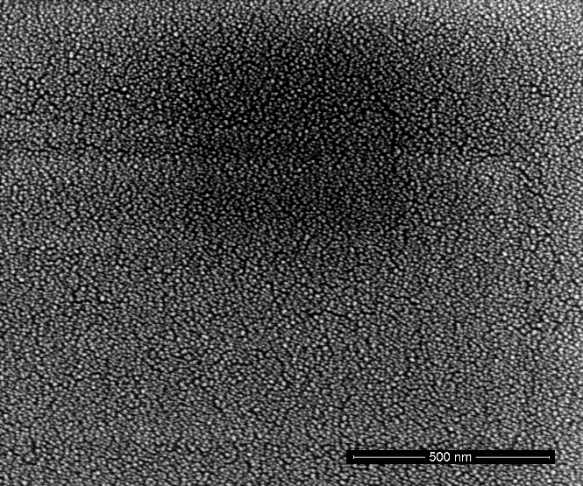

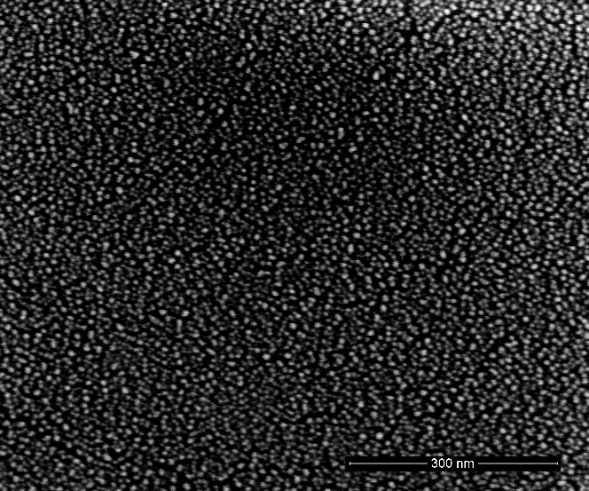


**Figure S2**. SEM micrographs of the as-deposited Cu film with evaporation rate of 0.1 nm/sec. Top view-different magnification.


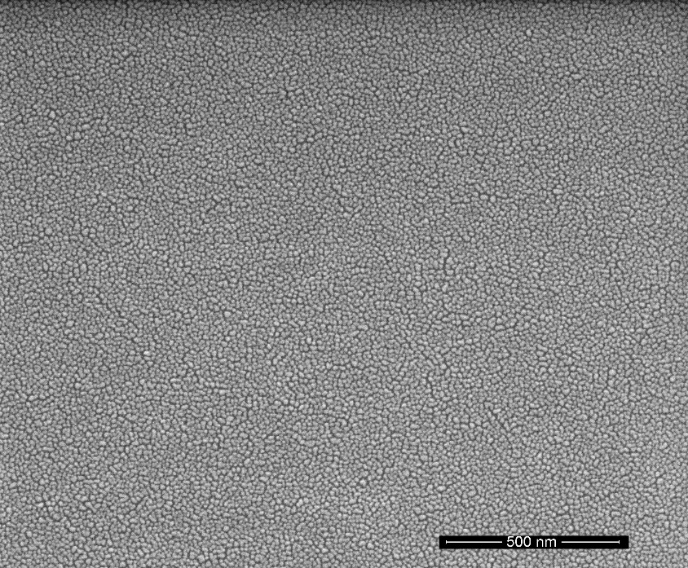


**Figure S3**. SEM micrographs of the as-deposited Cu film with evaporation rate of 3 nm/sec. Top view.


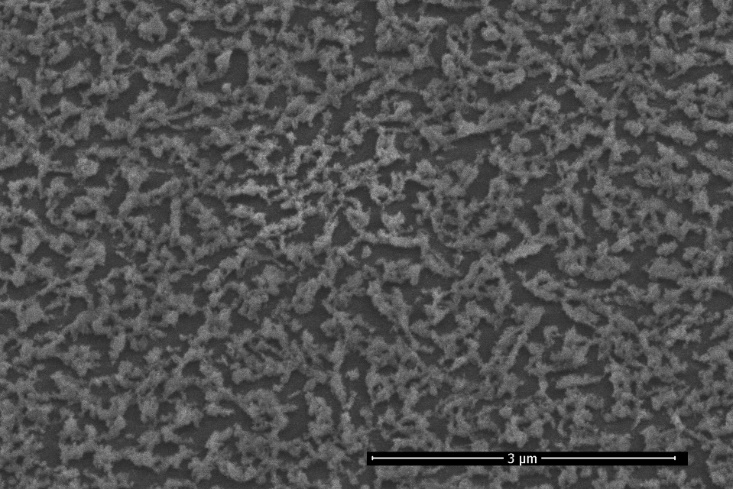


**Figure S4**. SEM micrographs Cu nanoporous film obtained with N2 plasma from the sample deposited at 3 nm/sec (after two repeated evaporations+plasma etching).


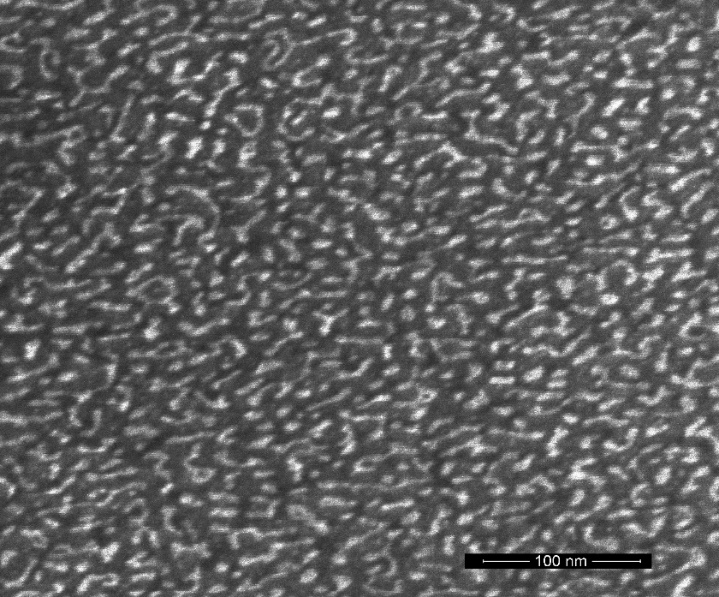


**Figure S5**. SEM micrograph of CuOx nanostructures obtained with O2 plasma etching. The quality of the image is limited by the low conductivity of the metal oxide.


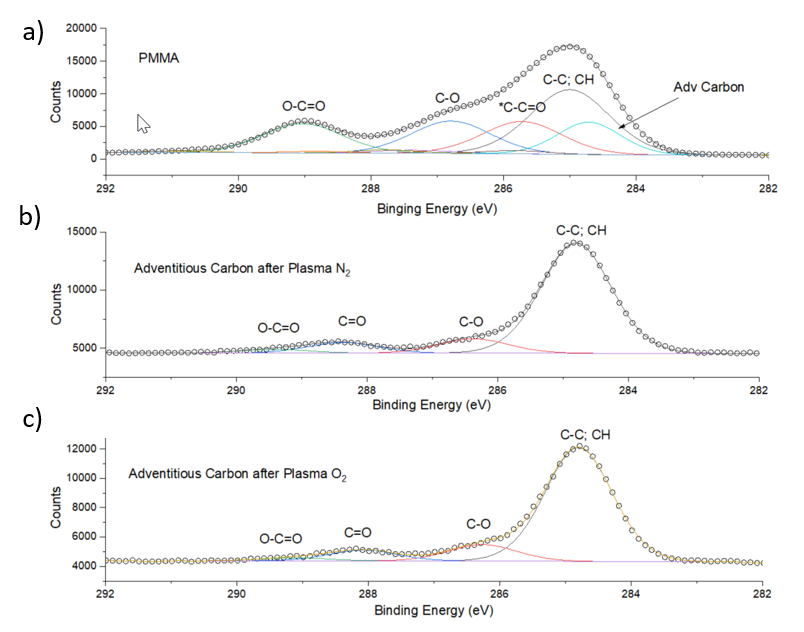


**Figure S6**. XPS analyses of the carbon peaks to demonstrate the complete removal of PMMA both after N_2_ and O_2_ plasma etching: (a) Cu film on PMMA – initial preparation step; (b) Cu film after N_2_ plasma etching; (c) Cu film after O_2_ plasma etching.

For XPS analyses, the Cu film is deposited on Si substrate and resulted, in all the cases, covered by a thin adventitious carbon film, a thin layer of carbonaceous materials due to atmospheric exposure. The C1s spectrum of PMMA+Cu sample gave consistent peak-shape results with the polymer structure.

After Plasma, both using O_2_ and N_2_, the PMMA is fully removed from the samples and C1s peaks are consistent with the adventitious Carbon found in the pristine Cu film.


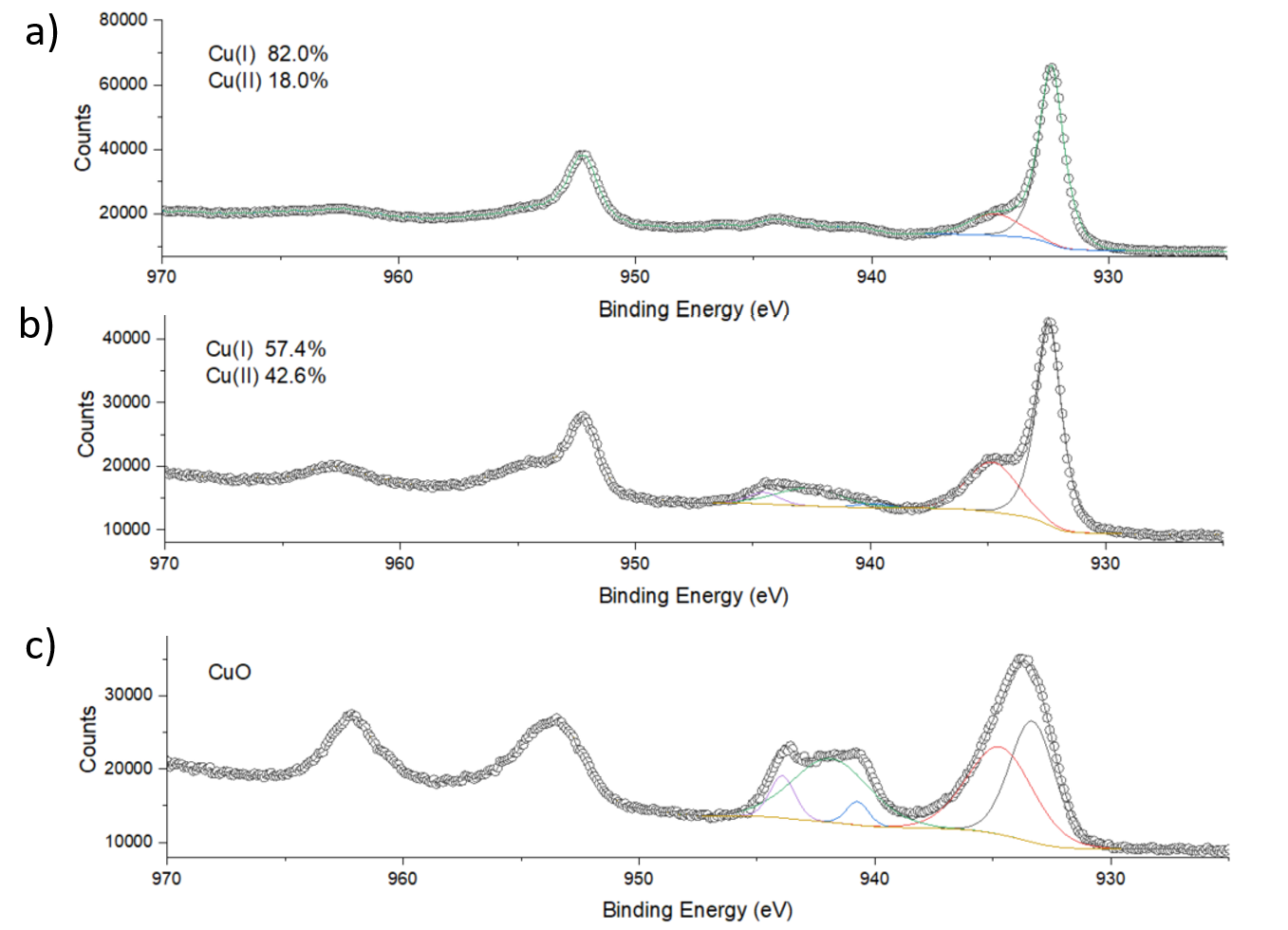


**Figure S7**. XPS analyses of the Cu peaks: (a) Cu film on PMMA – initial preparation step; (b) Cu film after N_2_ plasma etching; (c) Cu film after O_2_ plasma etching.

The Cu 2p spectrum shows a mixture of Cu(I) and Cu(II), mainly Cu(OH)_2_ , in the pristine Cu+PMMA sample. After N_2_ plasma the Cu(II) content increases while after the O_2_ plasma the Cu layer is completely cover by CuO oxides.

**Supporting Note – 2**

| **Oscillator Type** | **A_i_** | **E_0_ (eV)** | **γ_i_ (eV)** |
| --- | --- | --- | --- |
| Gaussian | 0.093 | 1.921 | 1.064 |
| Gaussian | 0.027 | 2.118 | 0.338 |
| Gaussian | 0.029 | 3.127 | 0.497 |
| Gaussian | 0.068 | 2.667 | 0.736 |

To gain a first fundamental insight into the optical properties of the Cu NIs, spectroscopic ellipsometry was employed. This technique provides precise measurements of the film's dielectric function, allowing us to characterize the complex refractive index and thereby evaluate the plasmonic response across a broad spectral range.

**Table 1**: Parameters used to carry out the fit of the ellipsometrical angles.

The dielectric permittivity of the Cu NIs was calculated by modelling the ellipsometry measurements as a series of gaussian oscillators (Table 1), to obtain a Kramers-Kronig consistent dispersion. The imaginary part of all the gaussian oscillators used for the fitting procedure is provided in Eq. 1:

$Im\left\{ \varepsilon_{Gauss} \right\}=A_{i}e^{\left( -\frac{E-E_{0}}{\gamma_{i}} \right)}$ (1)

where E_0_ is the central energy of the i_th_ oscillator, γ its damping and A its amplitude. The imaginary part of the Tauc-Lorentz oscillators is, instead, provided in Eq. 2:[25]

$Im\left\{ \varepsilon_{Tauc-Lor.} \right\}=\frac{A_{i}E_{0}\gamma_{i}\left( E-E_{g} \right)^{2}}{\left( E^{2}-E_{0}^{2} \right)^{2}+\gamma_{i}^{2}E^{2}}\frac{1}{E}, E>E_{g}; Im\left\{ \varepsilon_{Tauc-Lor.} \right\}=0, E\leq E_{g};$ (2)

**Figure S8:** (a) Real (black curve) and imaginary (red curve, associated right axis) part of the effective dielectric permittivity of the Cu NIs. Measured (dots) and fitted (solid lines) ellipsometric angles Ψ (b) and Δ (c).


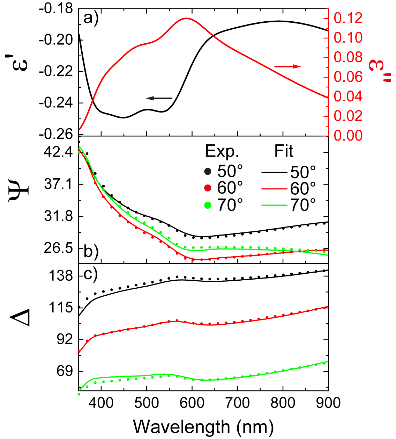


Here, the parameter E_g_ represents the optical band-gap. The associated real parts have been retrieved via Kramers-Kronig relations. The real and imaginary parts of the effective permittivity of the Cu NIs are shown in Figure S8 and are found to be very different from the homogenous film. Both the measured (dots) and fitted (solid lines) ellipsometrical parameters Ψ and Δ, collected at incidence angle θ_in_ = 50°, 60° and 70° are provided in Figure. The relatively low Cu fill-fraction, indeed, gives origin to a blend with a slightly metallic (< 1) effective dielectric permittivity with a low-loss Epsilon Near Zero character throughout the visible range.

As a reference layer, we measured the dielectric permittivity of a uniform Cu film. The results are shown in Figure S9a. To retrieve this dispersion, a standard model has been used based on Palik’s data.

As additional experiment, we also prepared the Cu NIs by using O_2_ plasma instead of N_2_. Following this procedure, we expected to obtain CuOx nanostructures. To verity it, we measured the dielectric permittivity of the film obtaining a good agreement with the tabulated CuO dielectric constants.


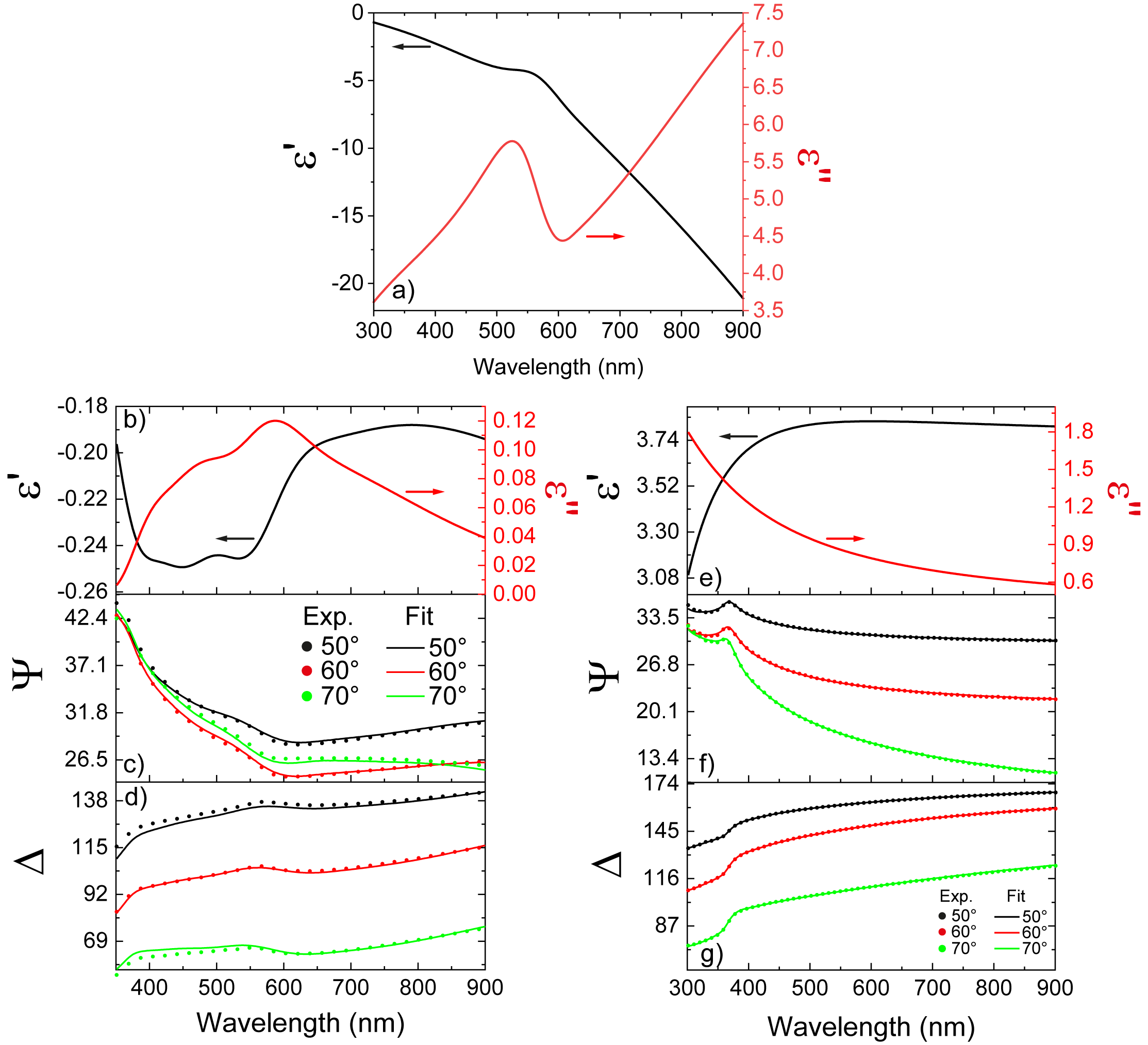


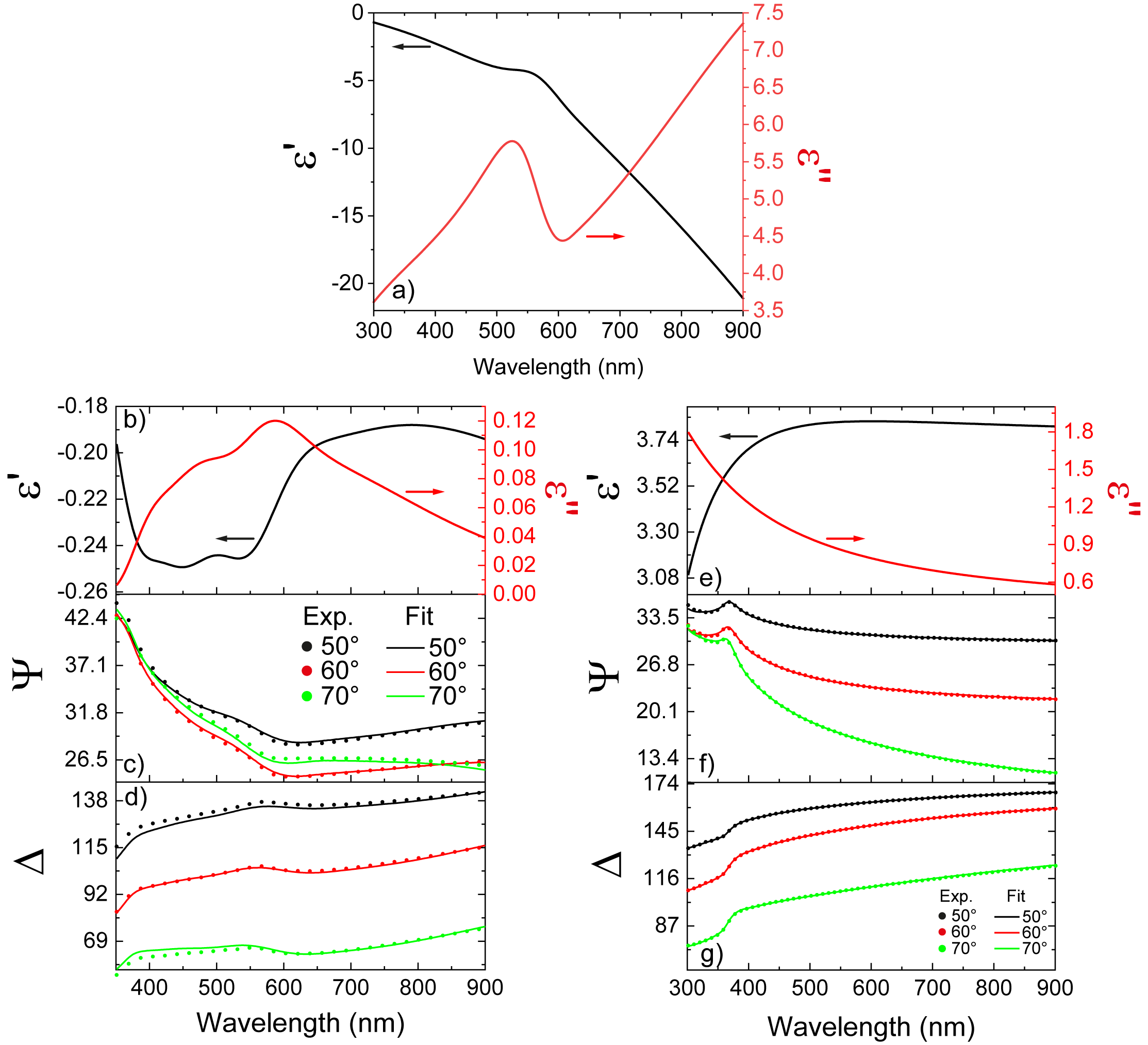


**Figure S9**: Real (black curve) and imaginary (red curve, associated right axis) part of the effective dielectric permittivity of (a) a deposited bare Cu film taken as a reference, (e) CuO nanoislands . Measured (dots) and fitted (solid lines) ellipsometric angles Ψ and Δ measured for CuO nanoislands (f,g).

The dielectric permittivity of CuO nanoislands is also shown in Figure S9e (black line for the real part and red line for the imaginary one). Also in this case, substantially different dielectric permittivity has been found. CuO is well-known for behaving as a dielectric in the visible range.^[2,3]^ A typical Cauchy-like dispersion has, therefore, been used for the real part of the refractive index *n(λ)* following the expression given in Eq. 3. The imaginary part *k(λ)* of the refractive index assumes the shape of a typical Urbach tail, whose expression is provided in Eq. 4:

$n\left( \lambda\right)=A_{n}+\frac{B_{n}}{\lambda^{2}}+\frac{C_{n}}{\lambda^{4}}$; (3)

$k\left( \lambda\right)=k_{amp}\cdot e^{g(E-E_{b})}$; (4)

Here, A_n_, B_n_ and C_n_ are variable fit parameters and determine the index dispersion. The values *k_amp_* and *g* are fitting parameters that determine the steepness of the absorbance curve. The band-edge parameter *E_b_* can be manually set, and it approximates the wavelength at which the Urbach tail begins to play a significant role.

In our model: *A_n_* = 1.94, *B_n_* = 0.02, *C_n_* = -0.002, *k_amp_* = 0.32, *g* = 0.44 and *E_b_* = 400 nm.

**Supporting Note – 3**


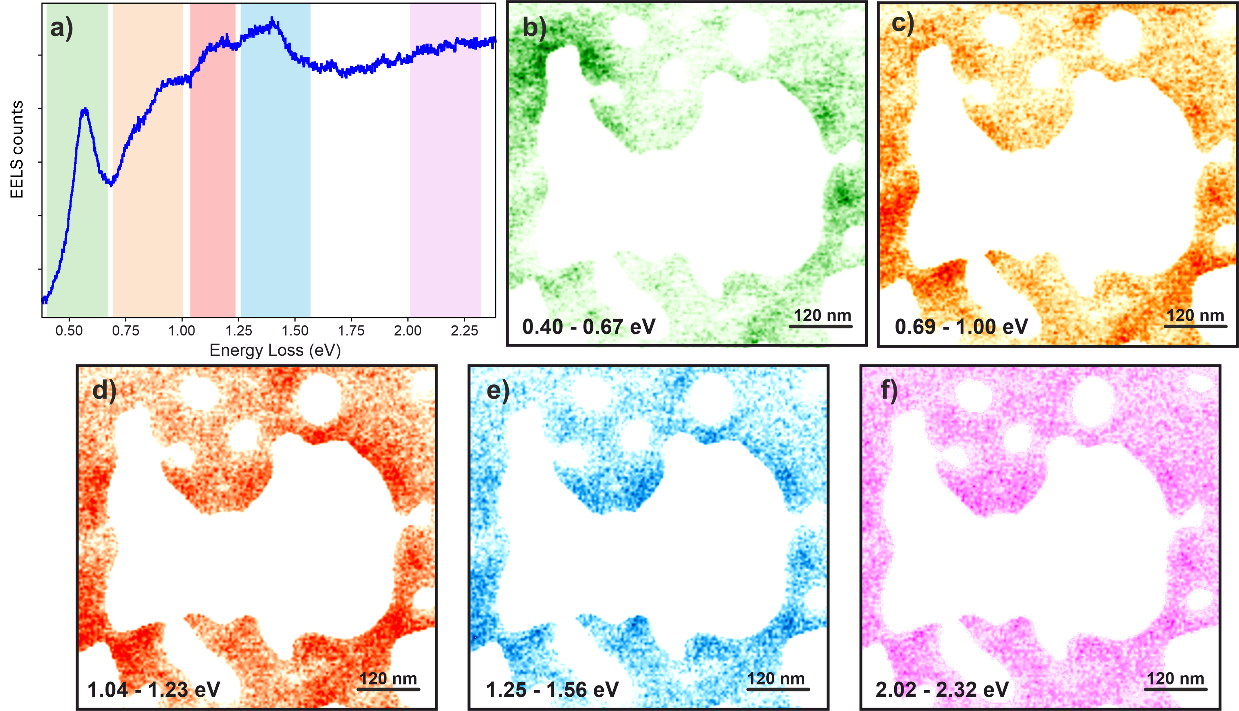


**Figure S10.** a) Integrated EELS signal across the entire area after background subtraction; b) to f) integrated intensities of the EELS signal within various energy windows. The width of the windows was chosen to be either the full width at half maximum of a Gaussian fit of the excitation or 0.3 eV (for panel f), since the signal isn’t as well defined as the other modes).

**Supporting Note – 4**


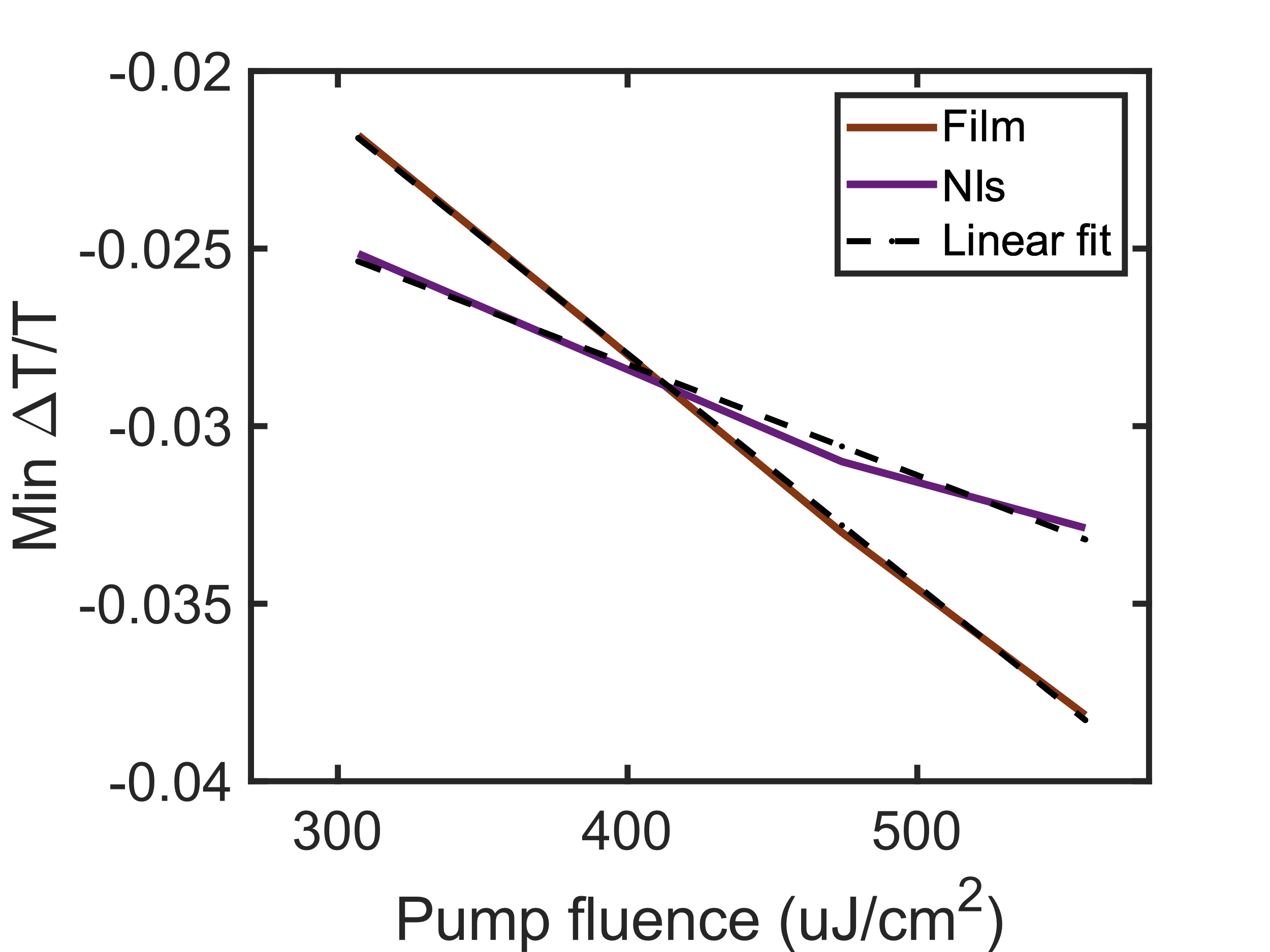


**Figure S11:** Fluence dependence. Pump fluence dependence of the Minimum transient transmission amplitude (maximum absolute value) at 577nm.

Figure S11 displays the measured minimum transient transmission amplitude at 577 nm as a function of varying pump fluences. The decreasing negative value with increasing fluence indicates that more carriers are excited when pump fluence is increased in both continuous and NIs film. Interestingly, they exhibit distinct linear relationships with pump fluence. The effective charge excitation in the Cu film experiences a greater increase with increasing photon intensity, owing to the NIs structure's lower filling factor compared to bulk Cu. This leads to a smaller absolute fluence dependence for NIs sample.


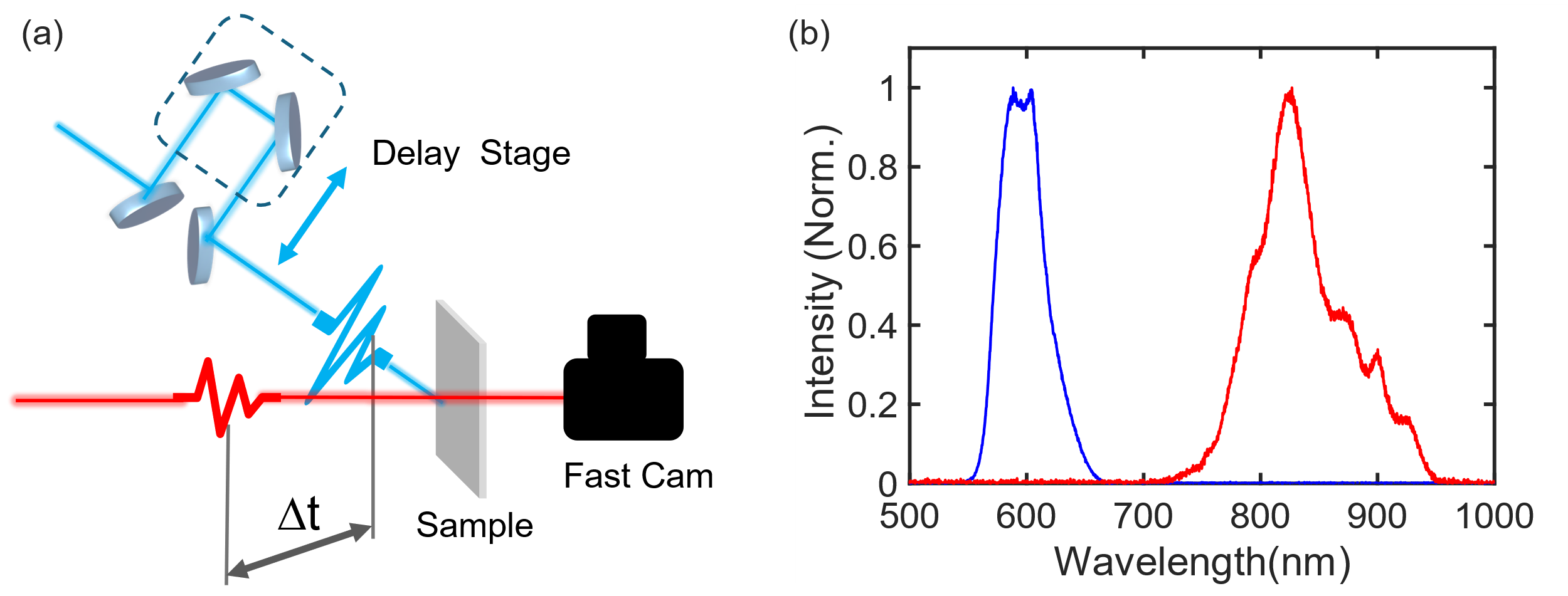


**Figure S12: The ultrafast pump-probe spectroscopy setup**. (a) Sketch of the setup, a delay stage is placed in the pump line to control the time delay (∆t) between pump and probe pulses. (b) The spectrum of the pump (red) and probe (blue) pulse employed in the measurements.

**Supporting Note – 5**


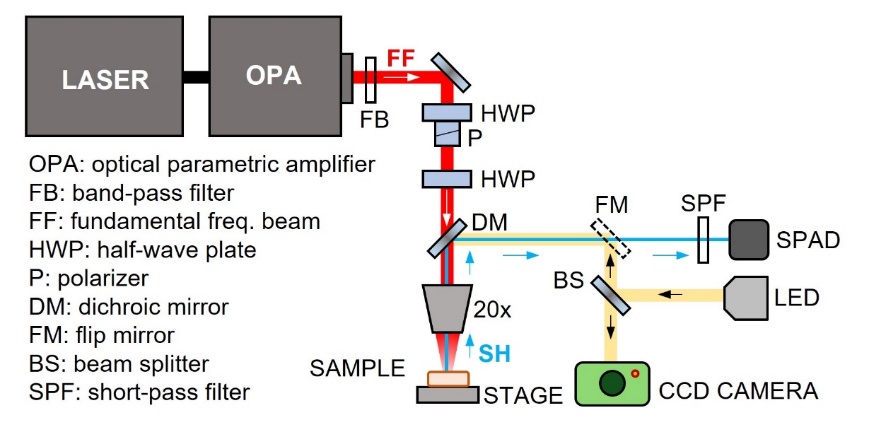


**Figure S13**. Sketch of the nonlinear microscopy setup


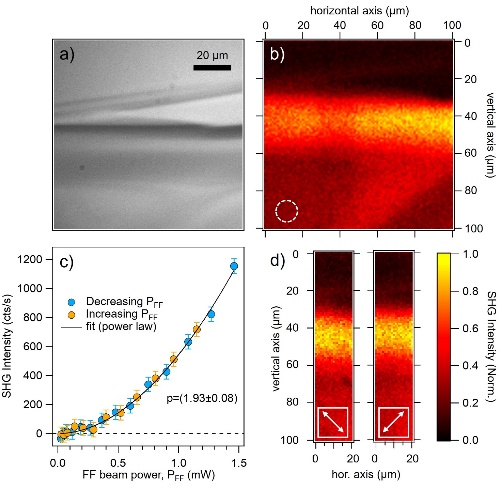


**Figure S14**. SHG response of Cu NIs. Space-resolved SHG scanning maps measured with FF beam with two orthogonal polarization directions (shown as insets).

**Supporting Note – 6**


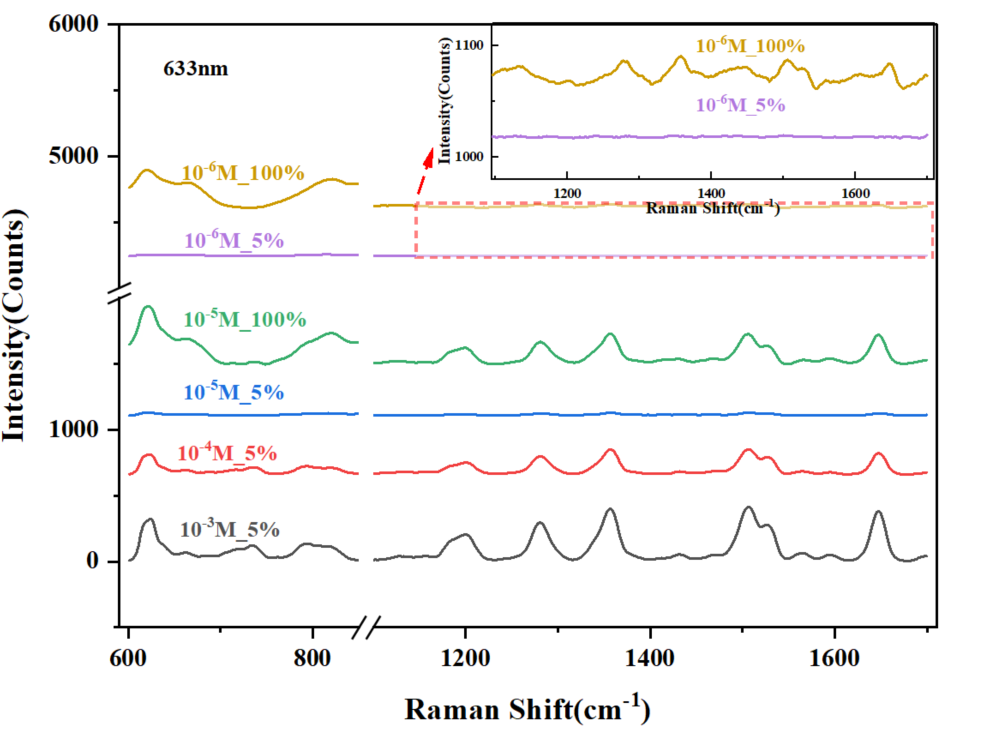


**Figure S15**. SERS spectra from R6G at different concentrations deposited on Cu-NIs.

**Supporting Note – 7**


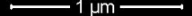

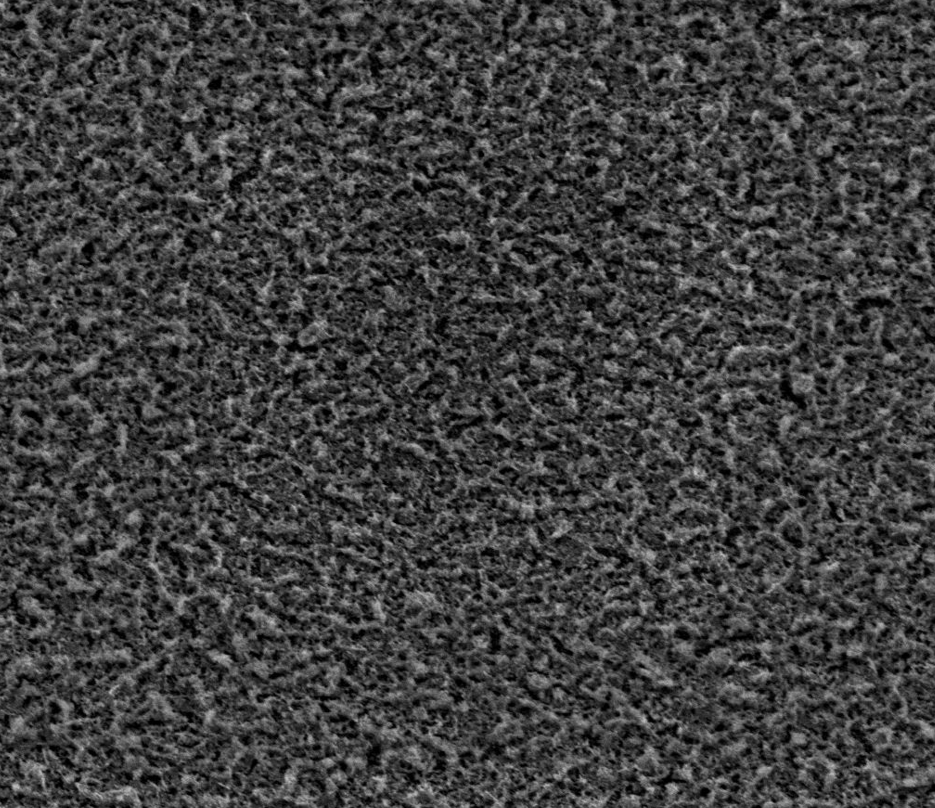


**Figure S16** . SEM micrograph illustrating the morphology of a bi-layer porous Au/Cu NIs film.


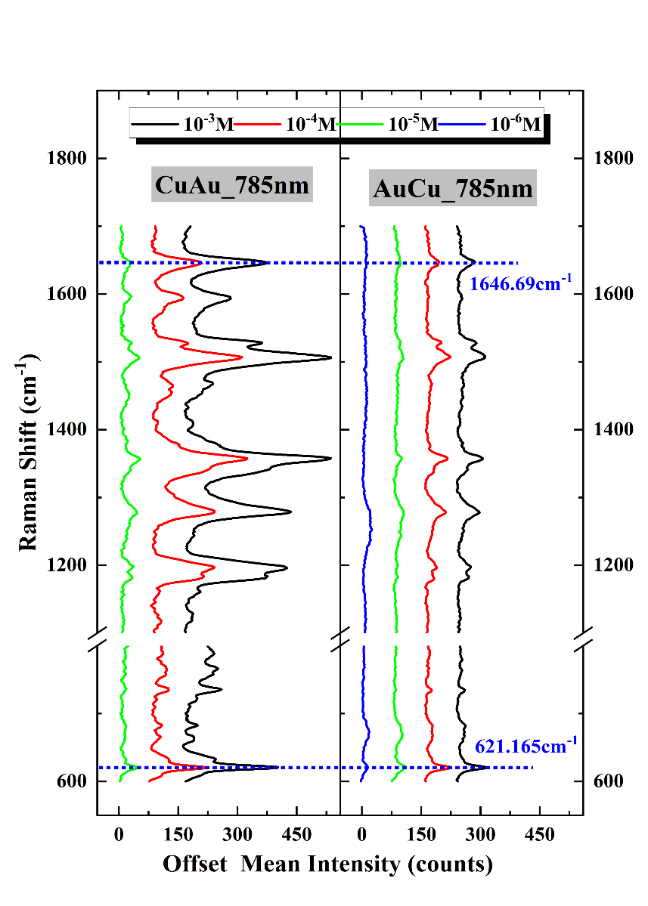


**Figure S17**. SERS spectra from R6G at different concentrations deposited on Au/Cu-NIs and on Cu-NIs/Au.
